# Rheological Performance of Asphalt Modified with Coal–Oil Co-Processing Residue

**DOI:** 10.3390/ma19091707

**Published:** 2026-04-23

**Authors:** Ruofei Qi, Jiuguang Geng, Pengju Huo, Yajie Guo, Wenhui Zhao, Yong Huang, Xiaoqian Zhang

**Affiliations:** 1 School of Materials Science and Engineering, Chang’an University, Xi’an 710018, China; ruofeiqi@163.com (R.Q.);; 2 The Northwest Research Institute of Chemical Industry Co., Ltd., Xi’an 710075, China; 3 School of Energy Science and Engineering, Henan Polytechnic University, Jiaozuo 454003, China; zhaowh@chd.edu.cn

**Keywords:** coal–oil co-processing residue, modified asphalt, asphalt mixture, rheological properties, reaction mechanism

## Abstract

To address high-temperature stability demands and promote resource utilization, this study investigates coal–oil co-processing residue (COCR) as an asphalt modifier. Penetration, softening point, ductility, rheological, and aging/storage evaluations were conducted on asphalt with varying COCR contents. Modification mechanisms were analyzed using FTIR, GPC, and SARA fractionation. The results revealed that COCR significantly enhanced high-temperature performance while slightly reducing low-temperature performance, showing good storage stability. At a 10% COCR content, the rutting factors of 70# and 90# asphalt increased by 44.8% and 46.2%, respectively, at 52 °C. Increased asphaltene content indicated that COCR reinforced the colloidal structure, thus improving the deformation resistance. At a 15% COCR content in mixture, the dynamic stability of asphalt mixtures increased by approximately 53.5% and 59.7% for 70# and 90# base asphalt, respectively. Considering overall performance balance, 10% COCR in 90# base asphalt would be recommended for regions with hot summers and warm winters.

## 1. Introduction

With the continuous growth in traffic demand and the increasing trend of vehicle axle loads, there is a pressing demand for enhanced load-bearing capacity in road infrastructure [1]. The high-temperature stability of asphalt pavement has become a critical factor affecting both pavement service life and traffic safety [2]. Therefore, enhancing the high-temperature performance of asphalt holds significant engineering importance [3,4].

To enhance the high-temperature performance of asphalt pavements, a range of modification techniques are routinely employed, each operating on distinct mechanisms [5]. Polymer modification, exemplified by Styrene–Butadiene–Styrene (SBS) and Ethylene–Vinyl Acetate (EVA) [6], fundamentally alters the asphalt binder by creating a continuous, reinforcing three-dimensional polymer network [7]. This network imparts significant elasticity and cohesion, effectively restraining asphalt flow and thereby drastically improving resistance to rutting [8]. Another prevalent method is crumb rubber modification, which incorporates ground waste tires [9]. The rubber particles swell upon absorbing light oil fractions from the asphalt, increasing binder viscosity and forming a resilient, particle-reinforced matrix that enhances both high-temperature stiffness and elastic recovery [10,11]. Additionally, the use of natural rock asphalt introduces a high concentration of asphaltenes and stabilizing minerals, which substantially harden the binder and boost its stability under heavy loads and elevated temperatures [12,13]. While these methods effectively improve high-temperature performance to varying degrees, they are often associated with persistent issues related to their production and disposal, such as high production costs, limited raw material availability, and potential environmental concerns [14,15].

In recent years, the application of industrial by-products in asphalt modification has gained considerable attention worldwide, driven by both economic and environmental considerations [16]. This paradigm shift represents a multi-faceted advancement; technically, it utilizes the unique physicochemical properties of by-products—such as their rigid microstructure, porosity, or surface activity—to enhance mechanical performance in ways that can rival those of traditional modifiers [17,18]. Environmentally and economically, it is a cornerstone of the circular economy, diverting waste from landfills, reducing the carbon footprint associated with both virgin material extraction and waste disposal, and offering a cost-effective raw material source [19,20]. Consequently, this approach not only addresses the technical requirements for performance enhancement but also aligns with the principles of circular economy and sustainable development [21], offering a profoundly promising and innovative technological pathway for advancing the next generation of sustainable asphalt pavement materials [22,23].

Meanwhile, while China’s coal abundance and oil scarcity have driven the rapid development of coal–oil co-processing, these characteristics generate hundreds of thousands of tons of low-value COCR annually, posing significant economic and environmental challenges [24,25]. As a type of solid waste, COCR contains abundant heavy components, which give it significant potential for enhancing the high-temperature performance of asphalt [26]. In recent research, multi-stage extraction has been explored to derive extracts from COCR for use as an asphalt modifier. Nevertheless, this process presents inherent challenges in terms of complexity and cost, which may ultimately hinder its scalability and widespread industrial adoption.

This study aims to systematically investigate the application potential of COCR-modified asphalt in enhancing the high-temperature stability of asphalt pavements. Asphalt binders with varying COCR contents were prepared, and a series of tests—including dynamic viscosity and short-term aging—were conducted to examine both the effects of different dosages on the high- and low-temperature properties of asphalt and performance changes before and after aging. A Dynamic Shear Rheometer (DSR) was employed to analyze the rheological properties of the modified asphalt, providing in-depth insights into the influences of different COCR incorporation ratios on its rheological behaviors. Storage stability tests were carried out to evaluate the performance evolution of modified asphalt during long-term storage. Microscopic characterization techniques, including FTIR, GPC, and four-fraction composition analysis, were utilized to investigate the modification mechanism of COCR on asphalt at the molecular level. Finally, the pavement performance of each modified asphalt mixture was assessed to validate the suitability of COCR-modified asphalt for road applications.

## 2. Raw Materials and Test Methods

### 2.1. Raw Materials

#### 2.1.1. Basic Property Analysis of COCR

The COCR used in this study was sourced from Shaanxi Yanchang Petroleum (Group), Xi’an, China. Its specific properties and technical indicators are presented in Table 1 and Table 2.

Both COCR and petroleum asphalt were derived from oil-related feedstocks and shared similar compositional characteristics. The main components of the COCR included residual oil, asphaltenes, unreacted coal powder, inorganic minerals (ash) from the raw coal, a small amount of coke (the carbonaceous solid residue), and catalyst, which contains some heavy metal content. As there are currently no specifications regarding heavy metal content in road asphalt, the standard “Control standards of pollutants in sludge for agricultural use” (GB 4284-2018) [27] was referenced to assess potential environmental contamination. The heavy metal content in the COCR was found to be significantly lower than the limits stipulated by this specification.

**Figure 1:**
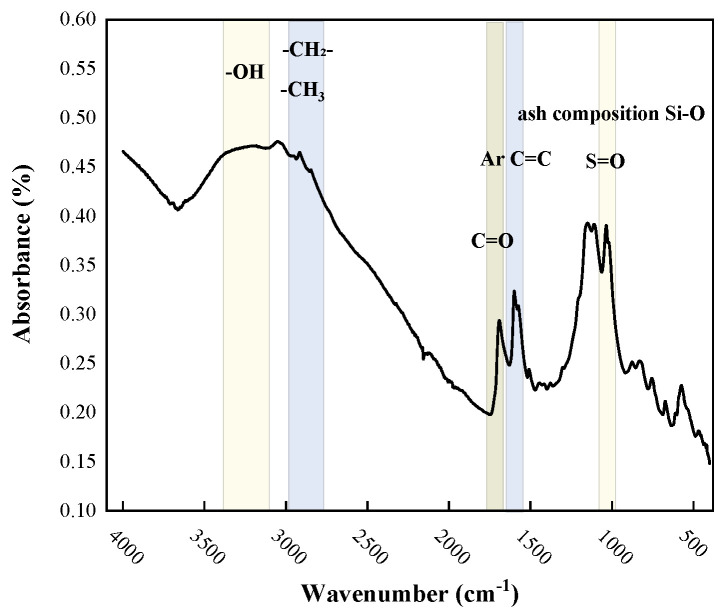
FTIR Results of COCR.

The chemical structure of the coal–oil co-processing residue (COCR) was characterized using FTIR, and the results are shown in the Figure 1 below.

Spectral analysis indicates that the residue is a complex organic mixture composed of aliphatic structures, aromatic structures, and various oxygen-containing functional groups.

The broad absorption band observed in the 3200–3400 cm^−1^ region is attributed to the stretching vibrations of O-H groups engaged in a complex network of hydrogen bonds. These interactions primarily include intra- and intermolecular hydrogen bonding between carboxylic acid groups, associations between hydroxyl groups and ether oxygen atoms, as well as hydrogen bonds between carboxyl groups and the aromatic system. These diverse hydrogen-bonding configurations collectively contribute to the characteristic broad spectral feature. The characteristic absorption bands observed in the 2800–3000 cm^−1^ region are attributed to the C-H stretching vibrations of methyl and methylene groups. This provides direct spectroscopic evidence for the presence of aliphatic moieties within the COCR, indicating that the residue retains substantial aliphatic carbon chains and hydroaromatic structures. The FTIR spectrum reveals two diagnostic peaks: the distinct absorption at 1600 cm^−1^ assigned to the skeletal vibrations of aromatic rings confirms the highly aromatic nature of COCR. Concurrently, the strong peak at 1700 cm^−1^ is attributed to the C=O stretching vibration, providing direct evidence for the presence of oxidized functionalities, such as ketones, which likely formed during co-processing. These structural features are crucial for understanding its modification mechanism on asphalt. The absorption peak at 1033 cm^−1^ can be primarily assigned to the S=O stretching vibration of sulfoxide groups, which is supported by the significant sulfur content detected via elemental analysis. Concurrently, silicate components present in the ash content of the COCR residue may also contribute a broad absorption band in this spectral region.

In summary, the FTIR analysis reveals that the COCR has a complex chemical composition. Its molecular structure is based on a framework of polycyclic aromatic hydrocarbons (PAHs) connected with or bridged by long-chain aliphatic structures, and it contains a considerable number of oxygen-containing functional groups.

#### 2.1.2. Base Asphalt Properties

The base asphalt used in this research consisted of 70# A-grade and 90# A-grade base asphalt. The specific technical indicators are listed in the Table 3 below.

#### 2.1.3. Preparation of Coal–Oil Co-Processing Residue Modified Asphalt

COCR was used as a modifier to prepare the modified asphalt. Since the addition of COCR can lead to a reduction in the low-temperature performance of asphalt, its dosage should not be excessively high.

The experimental design was established using 70# and 90# base asphalt separately as follows: COCR was added at mass ratios ranging from 9% to 16% relative to the base asphalt in increments of 1%. The control groups consisted of the unmodified 70# and 90# base asphalt [28].

### 2.2. Test Methods for Coal–Oil Co-Processing Residue Modified Asphalt

#### 2.2.1. Conventional Performance Tests of Modified Asphalt

In accordance with the “Test Methods for Asphalt and Asphalt Mixtures in Highway Engineering” (JTG E20-2011) [29], the three key indicators (penetration, softening point, and ductility) of the different modified asphalt binders were tested. Penetration was evaluated at 25 °C under a 100 g load for 5 s, the softening point was measured by the ring-and-ball method at a heating rate of 5 °C/min, and the ductility was tested at 15 °C with a stretching rate of 5 cm/min. Subsequently, the aging characteristics of the COCR-modified asphalt were investigated through the mass loss, retained penetration ratio, and post-aging ductility. The Rolling Thin Film Oven Test (RTFOT) was employed in this study to simulate the short-term aging of asphalt. Short-term aging was performed at 163 °C for 85 min.

Referring to the “Test Methods for Asphalt and Asphalt Mixtures in Highway Engineering” (JTG E20-2011), a segregation test (specifically, the softening point difference test) was performed on the COCR-modified asphalt. This test simulates practical conditions during transport and storage to evaluate the storage stability of the modified asphalt.

#### 2.2.2. Rheological Property Tests of COCR-Modified Asphalts

A Dynamic Shear Rheometer (DSR) (Anton Paar USA, Inc., Ashland, VA, USA) was used to conduct temperature sweeps, frequency sweeps, and Multiple Stress Creep Recovery (MSCR) tests on the COCR-modified asphalt specimens. These tests were performed to evaluate the high-temperature rutting resistance, high-temperature fatigue resistance, and relevant rheological properties of the modified binders [30].

The specific test parameters were as follows:

(1) Temperature sweep: Temperature range of 52 °C to 82 °C with an increment of 6 °C, applied strain of 1%, angular frequency of 10 rad/s, and oscillatory frequency of 1.6 Hz.
(2) Frequency sweep: Temperature range of 46 °C to 76 °C tested at twelve different frequencies of 0.1,0.2, 0.4, 0.6, 0.8, 1, 2, 4, 6, 8, 10, 20, and 30 Hz.
(3) MSCR Test: Test temperature of 64 °C.
(4) LAS Test: Test temperature of 25 °C.

#### 2.2.3. Modification Mechanism of COCR-Modified Asphalts

The modification mechanism was investigated using Fourier Transform Infrared (FTIR) (Thermo Fisher Scientific, Waltham, MA, USA) spectroscopy. Gel Permeation Chromatography (GPC) (Waters Corporation, Milford, MA, USA) was employed to analyze the molecular weight distribution of asphalt before and after modification. The compositional changes were studied through Four-Component Analysis (SARA analysis).

#### 2.2.4. Test Methods for Asphalt Mixtures with COCR-Modified Binder

The performance levels of asphalt mixtures prepared with the two base asphalts and their modifications at different COCR content levels were evaluated in terms of high-temperature stability, low-temperature crack resistance, and moisture susceptibility, following the Chinese specification “Test Methods for Asphalt and Asphalt Mixtures in Highway Engineering” (JTG 3410-2025) [31].

Firstly, the Marshall stability test (T0709-2025) was employed to assess the high-temperature stability of the mixtures. This test determines two key parameters: the Marshall stability (MS, in kN) and the flow value (FL, in 0.1 mm). The MS represents the maximum resistance of a compacted asphalt mixture specimen against plastic deformation under a standard loading rate (50 mm/min), directly indicating its ability to resist rutting under high temperatures. Conversely, the FL reflects the plastic deformation capacity of the mixture at the point of failure. An excessively high flow value suggests the mixture is too soft, compromising its high-temperature stability, while an overly low flow value indicates excessive brittleness. Furthermore, the rutting test (T0719-2025) was conducted to evaluate the high-temperature anti-rutting performance. The dynamic stability (DS, in cycles/mm) was calculated as the number of wheel passes required to produce 1 mm of rutting deformation under a 0.7 MPa load at 60 °C. A higher DS value indicates superior resistance to permanent deformation. Secondly, the water stability of the mixtures was evaluated using the immersion Marshall test (T0729-2025). This test compares the Marshall stability of specimens soaked in a 60 °C water bath for 48 h against those soaked for 30 min. The retained stability was calculated as the ratio of the stability after 48-h immersion to the standard stability, which served as a quantitative indicator of the mixture’s resistance to moisture damage. Finally, the low-temperature crack resistance was characterized using the small beam bending test (T0715-2025). This test, performed at −10 °C, determines the flexural–tensile strength, the maximum failure strain, and the flexural stiffness modulus of the mixture by applying a load at a constant rate of 50 mm/min to a prismatic beam specimen (250 mm × 30 mm × 35 mm). The failure strain, which is the ratio of the deflection at failure to the span, is the primary parameter for assessing low-temperature performance.

The AC-13 gradation was employed in this study. The optimum asphalt–aggregate ratio was used for all asphalt mixtures modified with different COCR contents. The coarse aggregate, fine aggregate, and mineral filler used in this study were all high-quality limestone. Their respective properties are presented in Table 4, Table 5 and Table 6 below.

## 3. Results and Discussion

### 3.1. Performance Tests of COCR-Modified Asphalt

#### 3.1.1. Conventional Performance Indicators

**Figure 2:**
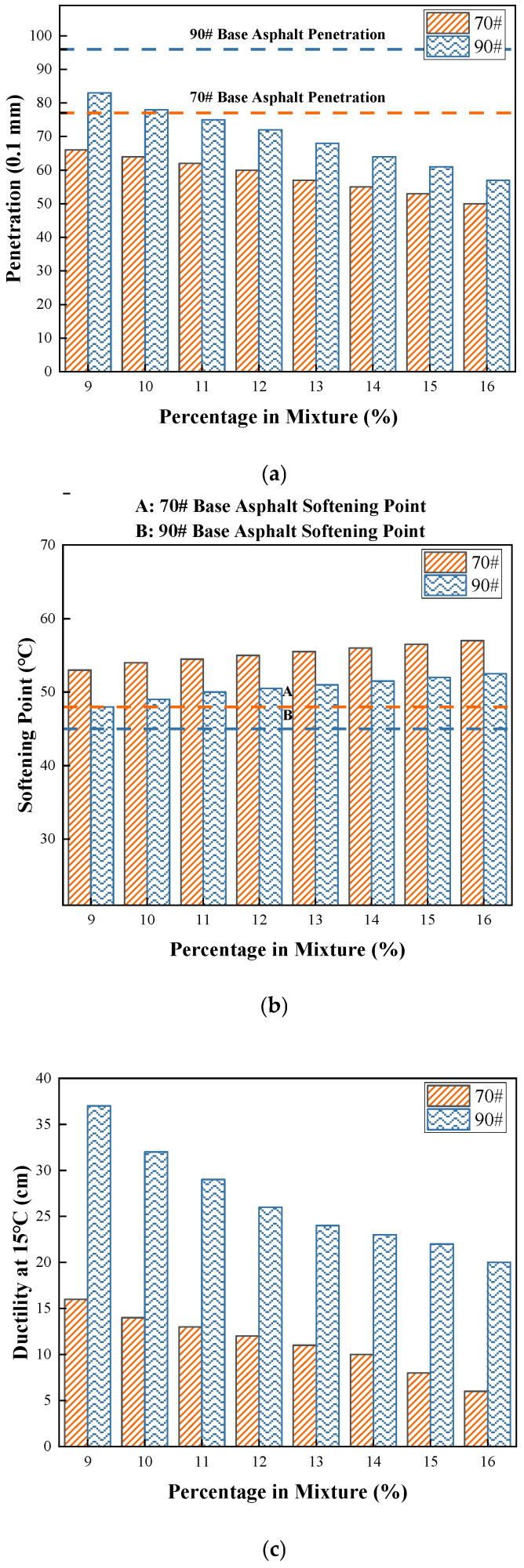
Conventional Performance Test Results of Modified Asphalt: (**a**) Penetration Test Results, (**b**) Softening Point Test Results, and (**c**) Ductility Test Results.

The conventional properties of modified asphalt, namely, penetration, ductility, and softening point, serve as fundamental indicators for evaluating its consistency, plasticity, and thermal susceptibility. These parameters provide a primary assessment of the binder’s performance grade and suitability for pavement applications. The results of the key physical parameters tests are presented in Figure 2 [32].

As shown in Figure 2, the incorporation of COCR leads to a decrease in penetration, an increase in softening point, and a reduction in ductility of the modified asphalt. This phenomenon can be attributed to the high asphaltene content present in the residue. The addition of COCR increases the viscosity of the asphalt binder and promotes a shift in its colloidal structure from a sol–gel type toward a more gel-like structure. Within the studied dosage range of up to 16%, the high-temperature stability of the modified asphalt exhibits a positive correlation with the COCR content.

#### 3.1.2. Aging Performance

##### Mass Change Rate

This study utilized the mass change rate of modified asphalt specimens subjected to short-term aging via the Rolling Thin Film Oven Test (RTFOT) to characterize the aging resistance of the binder. The control groups consisted of the unmodified 70# and 90# base asphalts.

**Figure 3:**
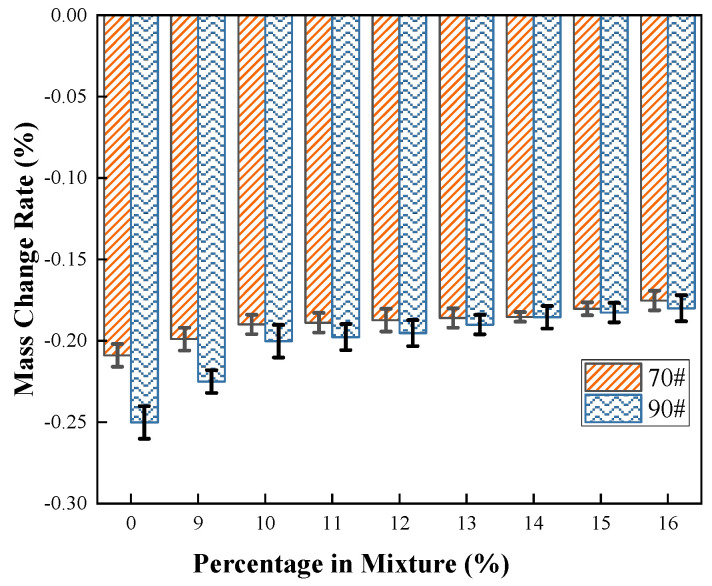
Mass Change Rate of Modified Asphalts.

Figure 3 indicates that the mass loss of the modified asphalt before and after RTFOT exhibits a negative correlation with the COCR content. At a COCR content of 16%, the mass change rates of the 70# and 90# base asphalts decreased by 20.1% and 27.7%, respectively. This phenomenon can be attributed to the high content of hard components within the COCR itself, which can absorb the light components in the asphalt, thereby reducing the mass loss of the modified binder. Furthermore, the COCR itself exhibits a relatively small mass loss during short-term aging. These two factors are the primary reasons for the observed reduction in the mass change rate of the modified asphalt.

##### Penetration Ratio

In this study, the effect of COCR on the anti-aging performance of asphalt was quantitatively evaluated by calculating the penetration retention rates of asphalt samples after short-term aging using the Rotating Thin Film Oven Test (RTFOT). The penetration ratio, defined as the percentage of the aged asphalt penetration to the unaged asphalt penetration, serves as an intuitive indicator reflecting the hardening degree and performance degradation trends of asphalt under the influence of heat and oxygen. Unmodified 70# and 90# base asphalts were used as control groups to systematically compare the variation patterns of the penetration ratio of modified asphalts with different COCR dosages.

**Figure 4:**
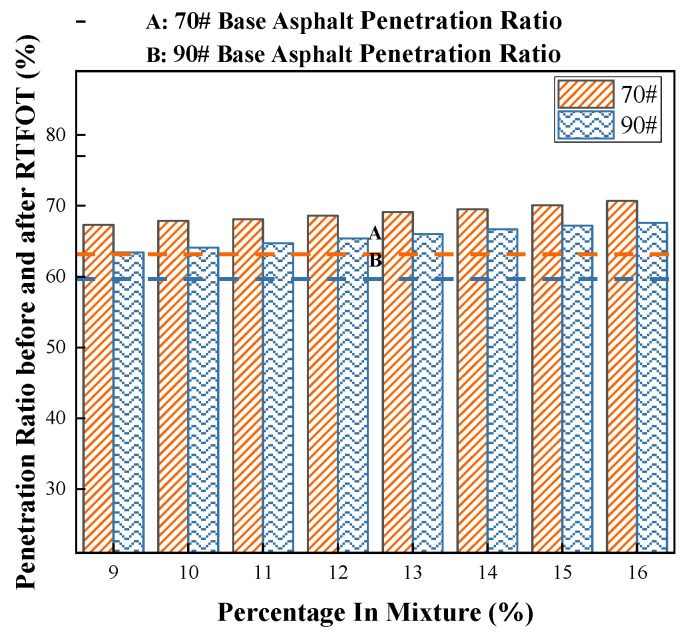
Penetration Ratio of Short-Term Aged Modified Asphalt.

Figure 4 shows that the penetration ratio of the modified asphalt after RTFOT increases with the COCR content. When the COCR content reaches 16%, the penetration ratios increase by 10% and 6% for the 70# and 90# base asphalts, respectively. This indicates that the incorporation of COCR results in increased hardening of the asphalt after aging, significantly enhancing its high-temperature stability and aging resistance.

#### 3.1.3. Storage Stability-Softening Point Difference

**Figure 5:**
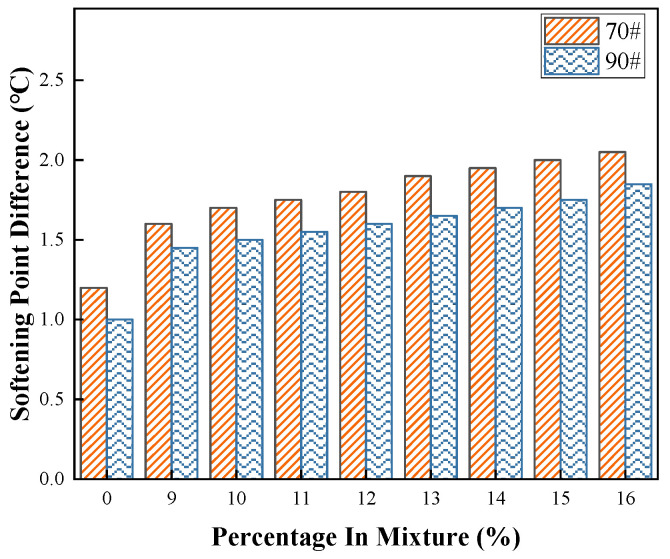
Softening Point Difference.

The presence of a significant amount of carbon powder within the coal–oil co-processing residue (COCR) makes it prone to segregation in asphalt, which can subsequently affect the construction quality of pavement using COCR-modified asphalt mixtures. Therefore, it is necessary to conduct storage stability tests on COCR-modified asphalt. Currently, the segregation test is the most widely used method for evaluating the storage stability of polymer-modified asphalt. This method primarily analyzes the difference in softening points between the top and bottom sections of the conditioned asphalt sample to investigate the storage stability of the modified binder. Following the standard “Test Methods for Asphalt and Asphalt Mixtures in Highway Engineering” (JTG E20-2011), four groups of samples (70# base asphalt, 90# base asphalt, and modified asphalts with different COCR contents) were subjected to high-temperature storage at 163 °C for 48 h. The test results are presented in Figure 5.

Figure 5 indicates that compared to the two base asphalts, the softening point difference of the modified asphalt with added COCR increases. The residue has the most significant impact on the storage stability of the 70# modified asphalt. At a 16% dosage, the softening point difference increases by 0.8 °C and 0.7 °C for the 70# and 90# asphalts, respectively, demonstrating a clear correlation between higher COCR content and larger softening point differences. During the blending process, components insoluble in asphalt, such as carbon powder from the COCR, settle at the bottom of the modified asphalt. This segregation leads to an increased softening point difference and consequently reduces the storage stability of the modified asphalt binder.

It should be noted that as COCR is not a polymeric material, it lies outside the scope of polymer-modified asphalt specifications. Consequently, based on the current Beijing technical specification for rock asphalt, whose properties it resembles, the modified asphalt is considered to have good storage stability if no separation is visually apparent in the test.

### 3.2. Rheological Properties of COCR-Modified Asphalt

#### 3.2.1. Temperature Sweep

Temperature sweep tests were performed using a Dynamic Shear Rheometer (DSR) with an angular frequency of 10 rad/s, covering a temperature range from 52 °C to 82 °C at 6 °C intervals. A 25-mm parallel plate geometry with a 1-mm gap was used, applying a strain level of 1% and a fixed loading frequency of 10 Hz.

**Figure 6:**
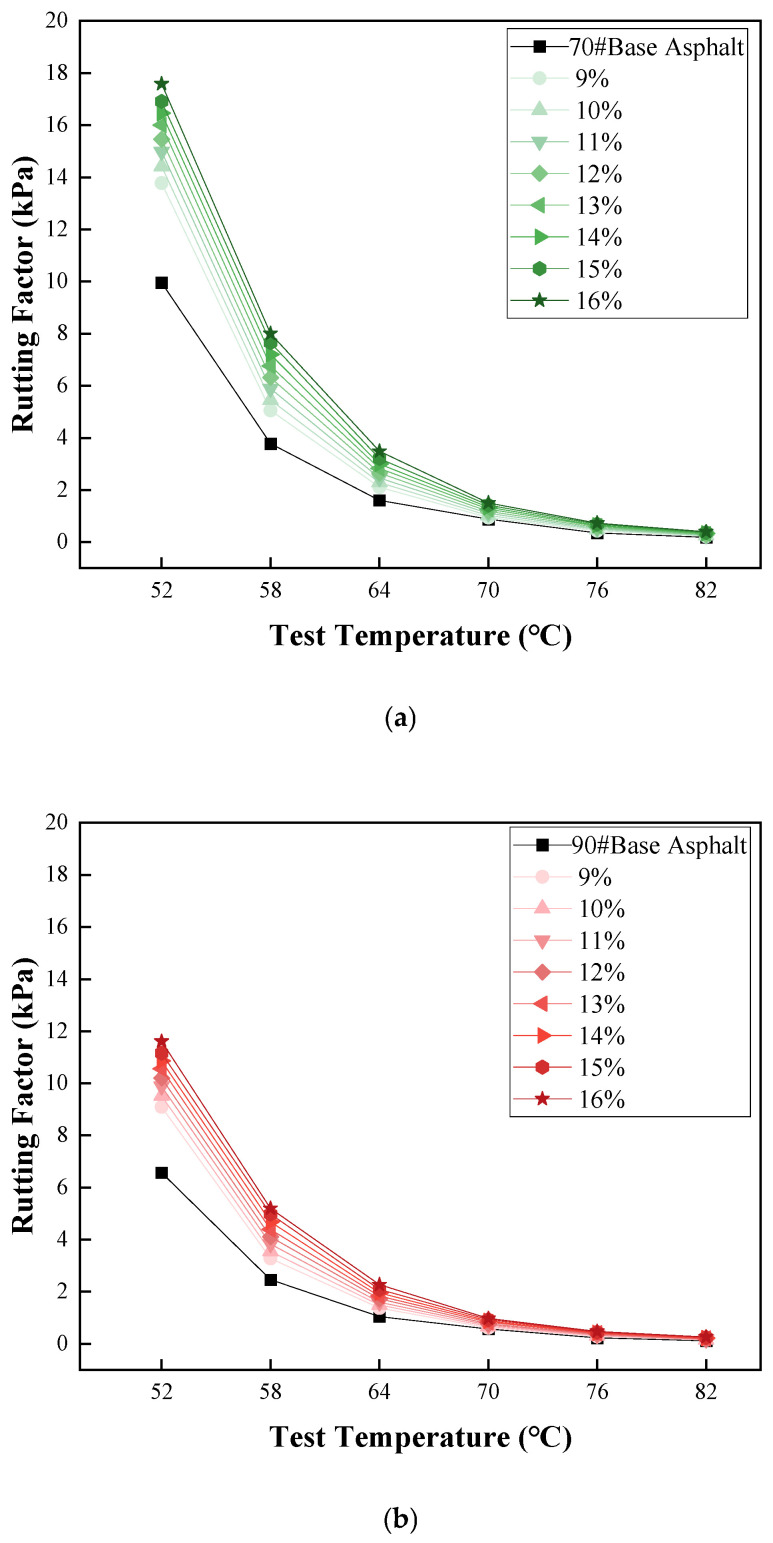
Rutting Factor: (**a**) 70# Asphalt and Its Modified Asphalt and (**b**) 90# Asphalt and Its Modified Asphalt.

Figure 6 presents the complex shear modulus and rutting factor results from the temperature sweep tests conducted on the COCR-modified asphalt.

As can be observed from Figure 6, the incorporation of COCR leads to fundamentally similar trends of change in both base asphalts. The addition of COCR increases the complex shear modulus and rutting factor of the asphalt. Compared to the base asphalts, the COCR-modified asphalts exhibit a higher rutting factor. Under a constant COCR content, the rutting factor of both the base and COCR-modified asphalts decreases with increasing temperature, indicating a decline in their high-temperature performance as temperature rises. After reaching a specific temperature, the rate of change in the rutting factor diminishes, eventually approaching zero. At a constant temperature, the rutting factor of the modified asphalt shows a positive correlation with the COCR content.

The degree of improvement in high-temperature performance provided by COCR is roughly similar for the different base asphalts. For instance, at a 16% dosage and 52 °C temperature, the rutting factor of the 70# asphalt increases from 9.9 kPa to 17.6 kPa (an improvement of approximately 77.8%), and the rutting factor of the 90# asphalt increases from 6.6 kPa to 11.6 kPa (an improvement of approximately 76%). The primary reason for this is that, compared with the base asphalts, COCR contains a higher proportion of heavy components such as asphaltenes. The addition of COCR promotes a shift in the asphalt’s colloidal structure from a sol–gel type toward a more gel-like structure [33].

#### 3.2.2. Frequency Sweep

As a complex viscoelastic material, the rheological properties of asphalt are not only influenced by temperature but also closely related to the loading frequency. This study utilized the preset frequency sweep test procedure of a Dynamic Shear Rheometer (DSR) to test and analyze asphalt binders with different coal–oil co-processing residue (COCR) contents. This approach aimed to obtain rheological indices of the asphalt samples under various loading frequencies and temperatures, enabling a comprehensive performance evaluation. Frequency sweep analysis was conducted on asphalts with different COCR contents using the 70# and 90# base asphalts as control groups. The frequency sweep tests were performed using a DSR with a 25-mm parallel plate geometry and a 1-mm gap, applying a strain level of 1%. The loading frequency range was from 0.1 Hz to 30 Hz, and the temperature range was from 46 °C to 76 °C at intervals of 6 °C.

**Figure 7:**
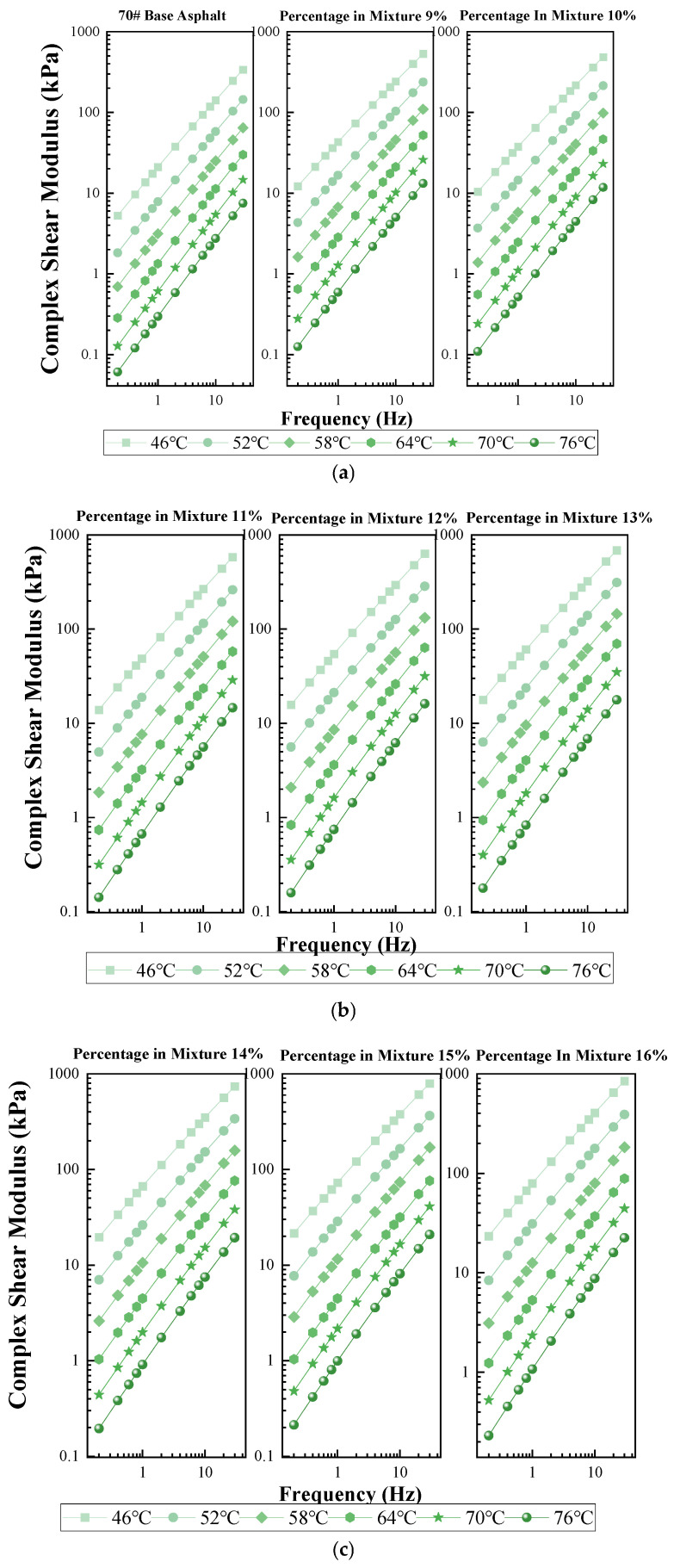

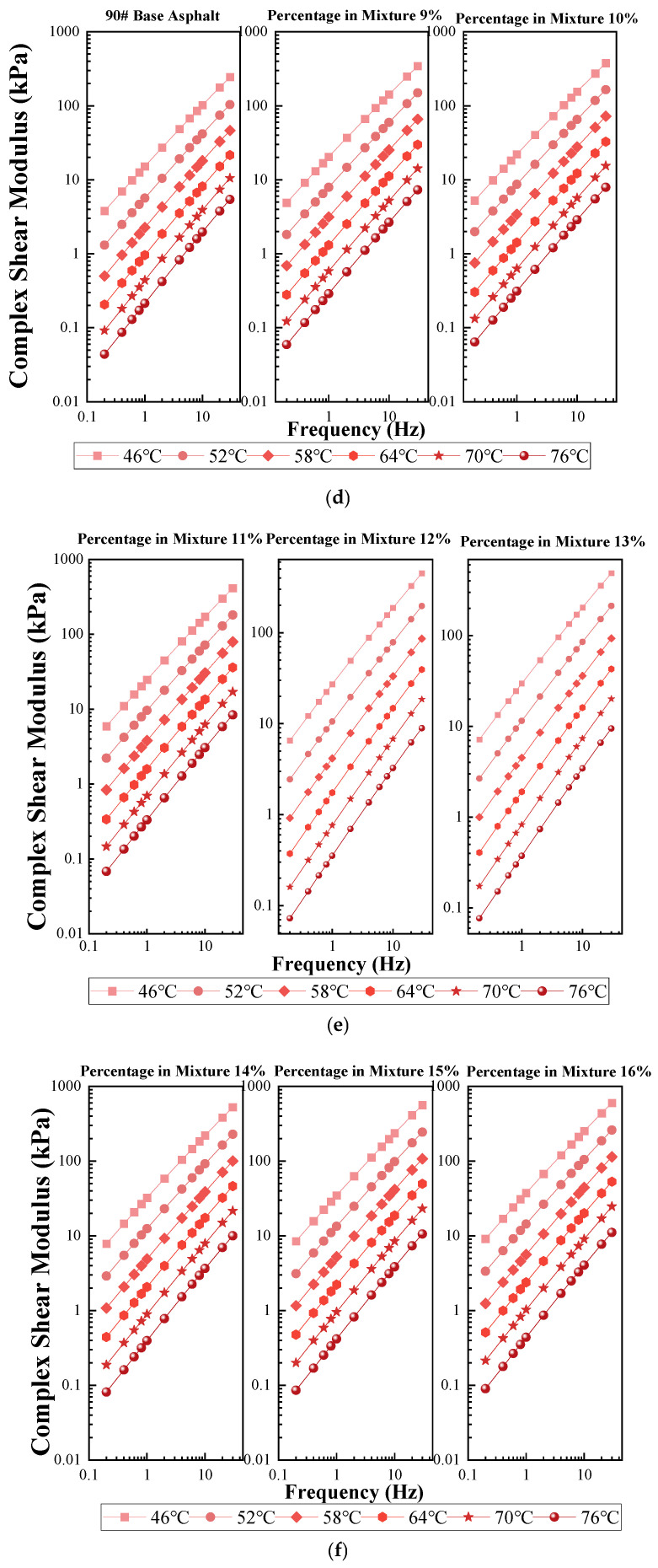
Frequency Sweep Test Results: (**a**–**c**) 70# Asphalt and Its Modified Asphalt and (**d**–**f**) 90# Asphalt and Its Modified Asphalt.

To describe the mathematical relationship between the complex shear modulus and the shear frequency, a double logarithmic coordinate system was employed for fitting. As shown in Figure 7, with increasing frequency, the elastic characteristics of the viscoelastic material become more dominant. Conversely, it exhibits characteristics more typical of a viscous fluid at lower frequencies. At the same test temperature, all asphalt samples show a similar trend: the complex shear modulus exhibits a positive correlation with the sweep frequency. This indicates that the asphalt displays deformation characteristics similar to those of an elastic body under the set shear loading frequencies. At the same test frequency, the complex modulus of the asphalt samples decreases as the test temperature increases. This suggests that the asphalt exhibits more viscous characteristics at higher temperatures.

Under identical test temperature and frequency conditions—for example, for the 90# base asphalt at 76 °C and 30 Hz—the complex shear modulus of the asphalt with a 16% COCR content is higher than that of the base asphalt, increasing from 5.41 kPa to 11.09 kPa. This demonstrates that the addition of COCR makes the elastic characteristics of the asphalt more prominent and enhances its resistance to deformation, thereby significantly improving the high-temperature stability of the asphalt.

This improvement is attributed to the high content of heavy components, such as asphaltenes and resins, in the COCR. These components lead to the hardening of saturates and aromatics within the modified asphalt. Furthermore, heavy components like asphaltenes and resins can reinforce the structural strength of the asphalt and enhance its viscoelasticity. Consequently, the modified asphalt is less prone to deformation under external stress at high temperatures, resulting in improved high-temperature stability [34].

#### 3.2.3. Multiple Stress Creep Recovery (MSCR)

This section employs the MSCR test to study the effect of adding COCR as a modifier on the elastic recovery capability of asphalt. The test temperature was set at 64 °C. Using a Dynamic Shear Rheometer (DSR), two stress levels, 0.1 kPa and 3.2 kPa, were applied to the asphalt specimens. The average percent recovery of the modified asphalt samples under these two different stress states was measured. The test results are presented in the figure below.

**Figure 8:**
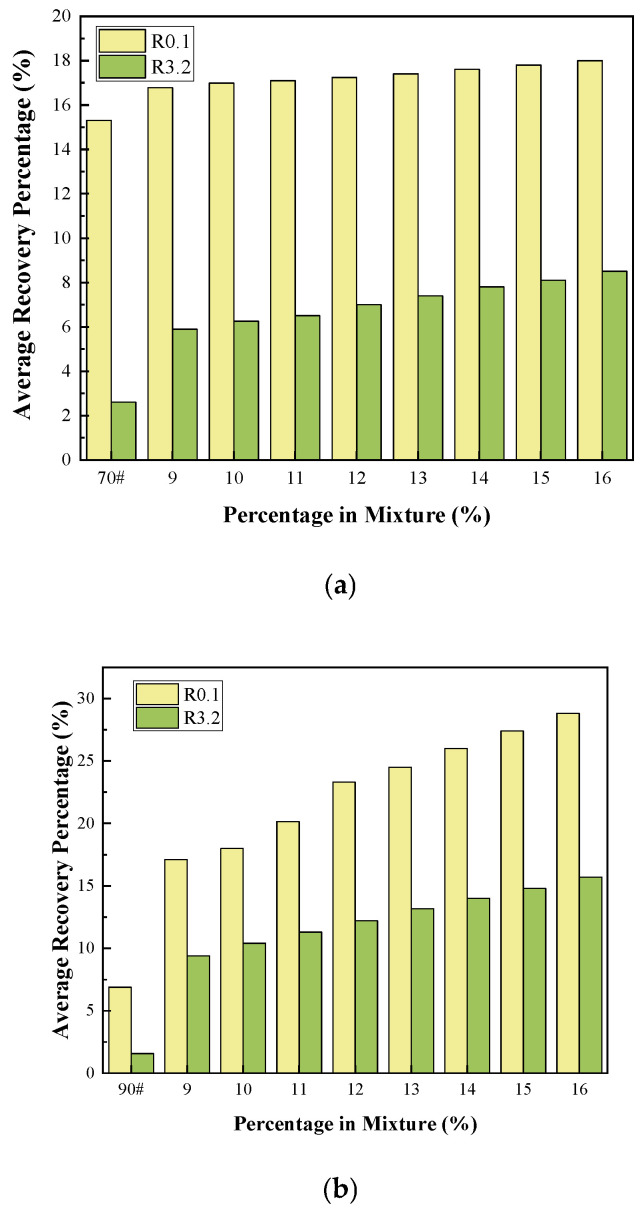
(**a**) 70# Asphalt Average Recovery Percentage Under Different Load Levels and (**b**) 90# Asphalt Average Recovery Percentage Under Different Load Levels.

**Figure 9:**
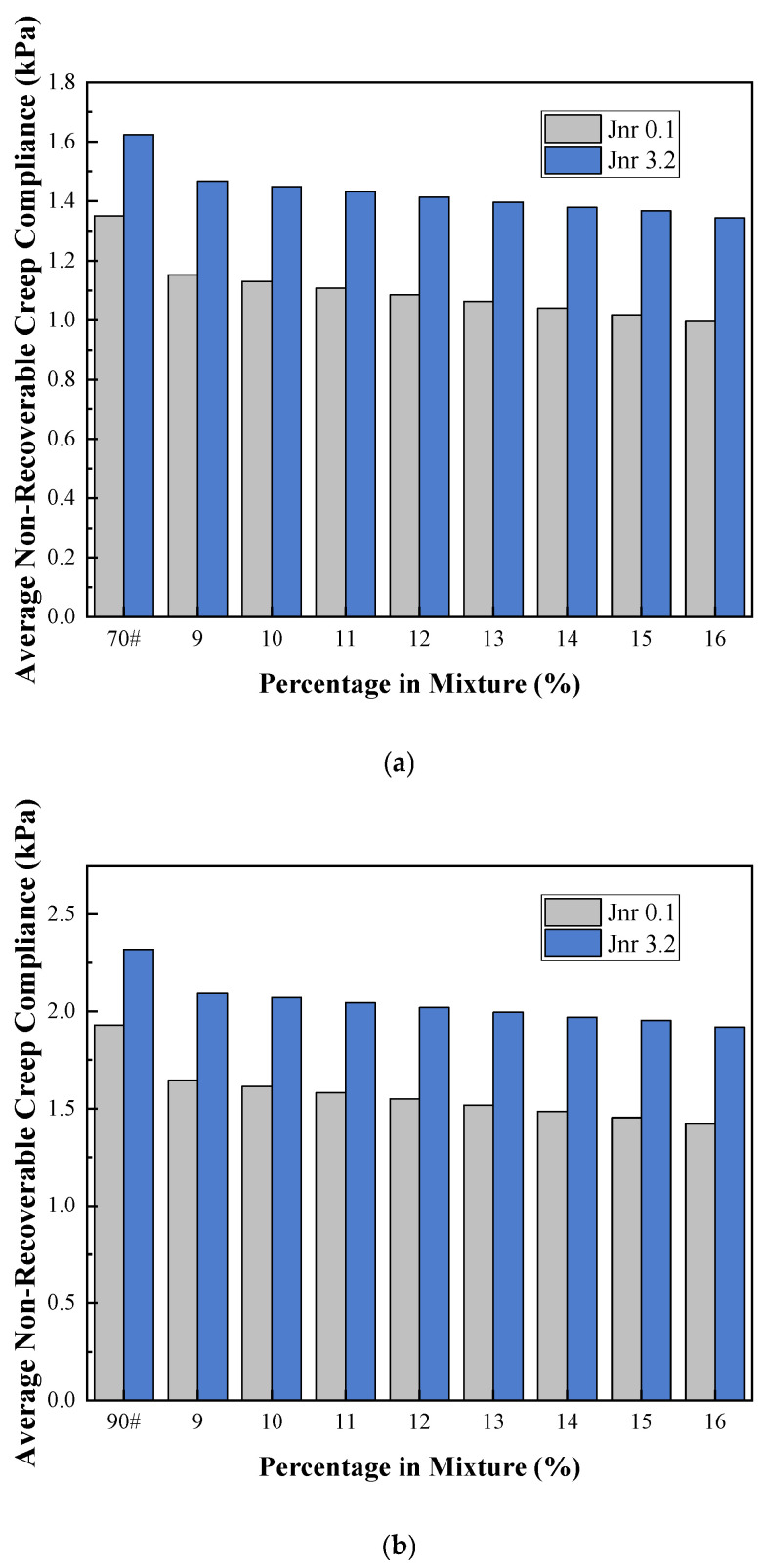
(**a**) Non-recoverable Creep Compliance of 70# Asphalt and (**b**) Non-recoverable Creep Compliance of 90# Asphalt.

As can be seen from Figure 8 and Figure 9 below, under the two different stress levels of 0.1 kPa and 3.2 kPa, the creep characteristics of the two base asphalt materials show little difference, with fundamentally similar trends of change. Judging from the difference in the non-recoverable creep compliance percentage, the addition of COCR leads to an increase in this value. This is because as the COCR content in the modified asphalt increases, the Jₙ significantly decreases when moving from the low stress condition (0.1 kPa) to the high stress condition (3.2 kPa). This indicates that incorporating a higher amount of COCR into the asphalt strengthens the internal structure of the modified binder, making it less prone to flow and permanent deformation under heavy loads, and its performance becomes less sensitive to stress variations. The increase in the average percent recovery directly indicates enhanced elasticity of the asphalt, a stronger resistance to permanent deformation, and a significant improvement in high-temperature rutting resistance [35].

#### 3.2.4. Linear Amplitude Sweep (LAS) Test

Under the coupled effects of long-term traffic loading and environmental factors, the internal structure of asphalt pavement is prone to forms of initial damage, such as microcracks and interlayer slippage. These microscopic defects continuously propagate and coalesce with an increasing number of load repetitions, eventually evolving into macroscopic cracks and leading to structural fatigue failure of the pavement. As a key constituent material of pavement structures, asphalt possesses fatigue resistance that directly determines the overall service life and durability of the pavement. Therefore, systematically investigating the influences of COCR on the fatigue characteristics of modified asphalt is of great significance for scientifically evaluating the engineering applicability of COCR.

**Figure 10:**
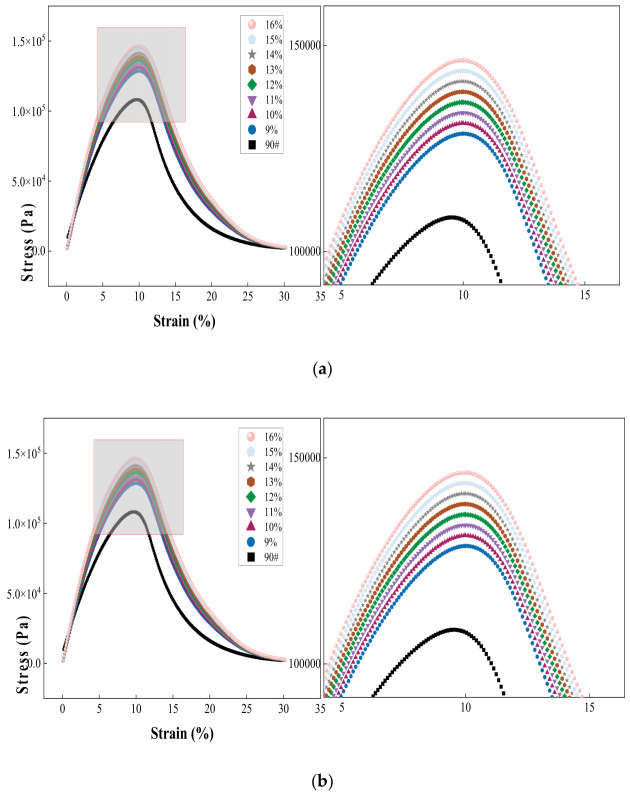
(**a**) LAS Test Results of 70# Asphalt and (**b**) LAS Test Results of 90# Asphalt.

As shown in Figure 10, which presents the LAS test results at 25 °C for 70# and 90# modified asphalts with different percentages in mixtures of COCR, it can be observed that for all asphalt specimens, the shear stress exhibits a pattern of first increasing and then decreasing with increasing strain, presenting a distinct peak stress point. When the strain is below the peak strain, the shear stress increases as the strain increases. When the strain exceeds the peak point, the shear stress begins to decrease, indicating the occurrence of irreversible damage within the material and its entry into the fatigue failure stage.

In terms of the change in critical strain amplitude (γ_max), with increasing percentage of COCR in the mixture, the critical strains of both modified asphalts did not change significantly, remaining essentially stable within the range of 9–11%. The critical strain of the 70# base asphalt was 10.4%, and it was 10.5% at a COCR percentage of 16% in the mixture; for the 90# asphalt, the critical strain changed from 9.6% to 9.9%. The essentially unchanged critical strain indicates that the incorporation of COCR does not alter the ultimate deformation capacity of the asphalt when it undergoes fatigue failure, and the maximum strain amplitude that the material can sustain before failure is primarily controlled by the inherent properties of the asphalt itself [36].

### 3.3. Microstructure and Modification Mechanism of COCR-Modified Asphalt

#### 3.3.1. Fourier Transform Infrared (FTIR) Spectroscopy Analysis

Fourier Transform Infrared (FTIR) spectroscopy was performed to analyze the COCR-modified asphalt, and the experimental results are shown in the figure below.

**Figure 11:**
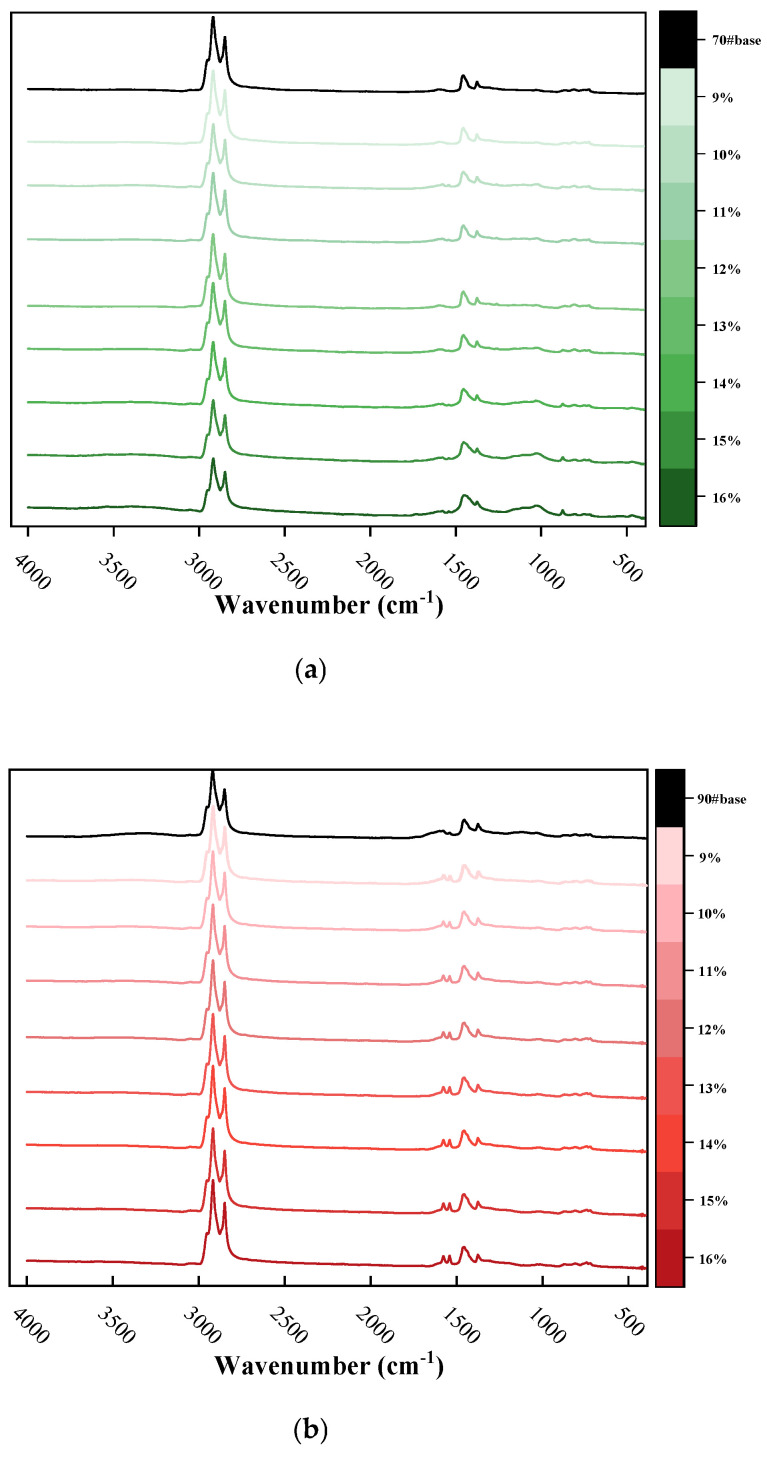

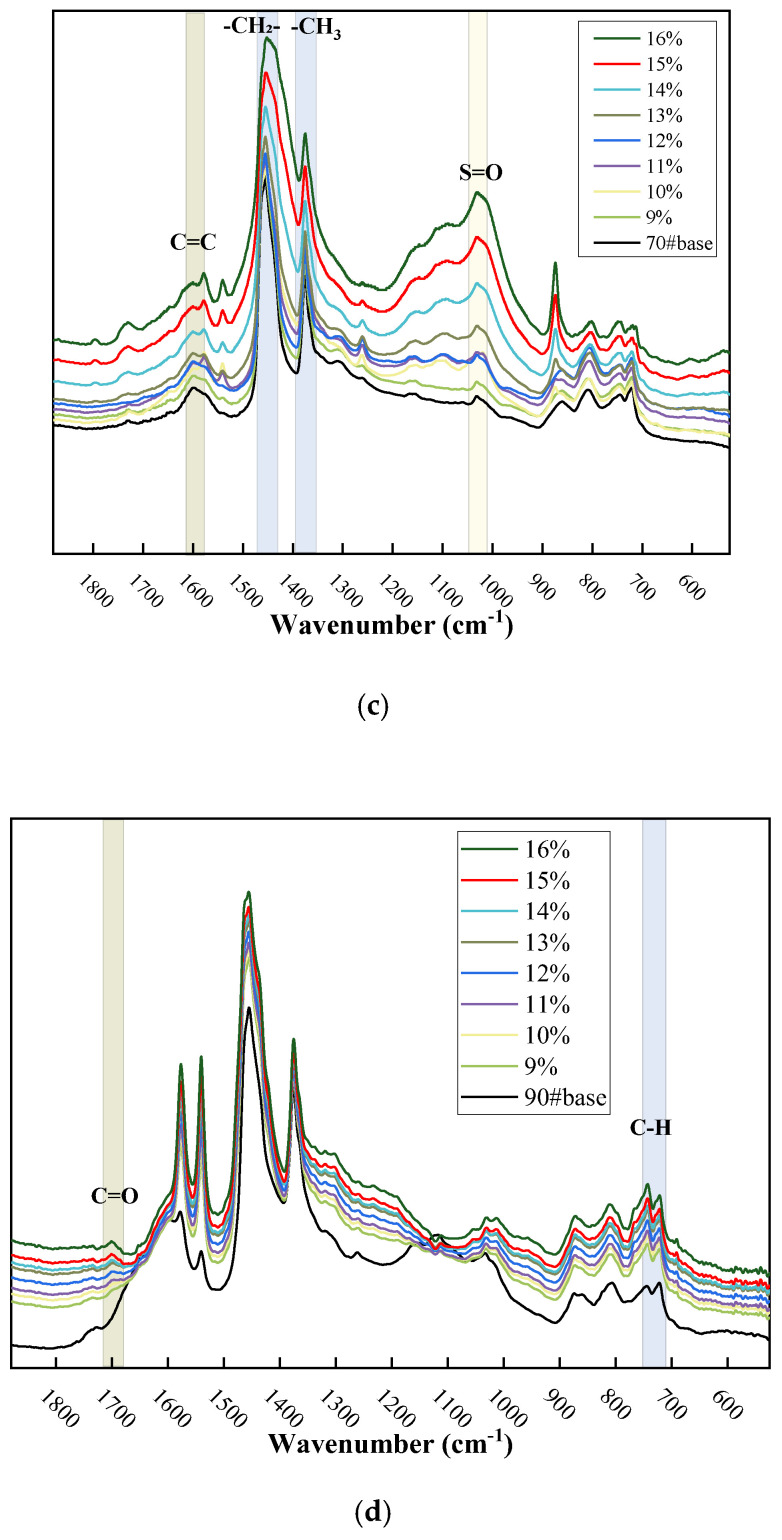
(**a**) FTIR Results of 70# Asphalt, (**b**) FTIR Results of 90# Asphalt, (**c**) FTIR Results of 70# Asphalt (Partial Enlarged View), and (**d**) FTIR Results of 90# Asphalt (Partial Enlarged View).

As shown in the Figure 11 below, the FTIR analysis indicates that all asphalt samples exhibit characteristic absorption peaks at 2924 cm^−1^ and 2852 cm^−1^. These are attributed to the stretching vibrations of -CH_2_ and C-H bonds, confirming the presence of saturates in the samples. The absorption peaks observed at 1450 cm^−1^ and 1373 cm^−1^ correspond to the C–H in-plane bending vibrations of -CH_2_ and -CH_3_, respectively, demonstrating the presence of alkyl groups in both the base and modified asphalt samples. Furthermore, the absorption band near 1600 cm^−1^ may originate from the stretching vibration of olefinic C=C bonds or carbonyl C=O vibrations. This feature indicates the presence of aromatic compounds in the samples. These findings are consistent across both the base asphalts and the COCR-modified asphalts [37].

It is noteworthy that a new absorption peak emerges at 1035 cm^−1^ in the modified asphalt sample. This is attributed to Si–O bonds from the ash content in COCR, with a potential contribution from sulfoxide (S=O) groups. The absorption peaks at 1375 cm^−1^ and 1450 cm^−1^ are due to the combined vibrations of -CH_2_- and -CH_3_ groups. The absorption peak at 1700 cm^−1^ is assigned to the carbonyl (C=O) stretching vibration, which is indicative of carboxylic acids and/or ketones. This suggests that an oxidation reaction may have occurred during the modification process of the asphalt.

Based on the experimental results, no significant new absorption peaks can be observed in the COCR-modified asphalt, indicating that the modification mechanism is not chemical in nature. Instead, the performance changes are achieved by introducing the inherent heavy asphalt components present in COCR, which physically influence the properties of the base asphalt.

#### 3.3.2. GPC Test Results of COCR-Modified Asphalt

**Figure 12:**
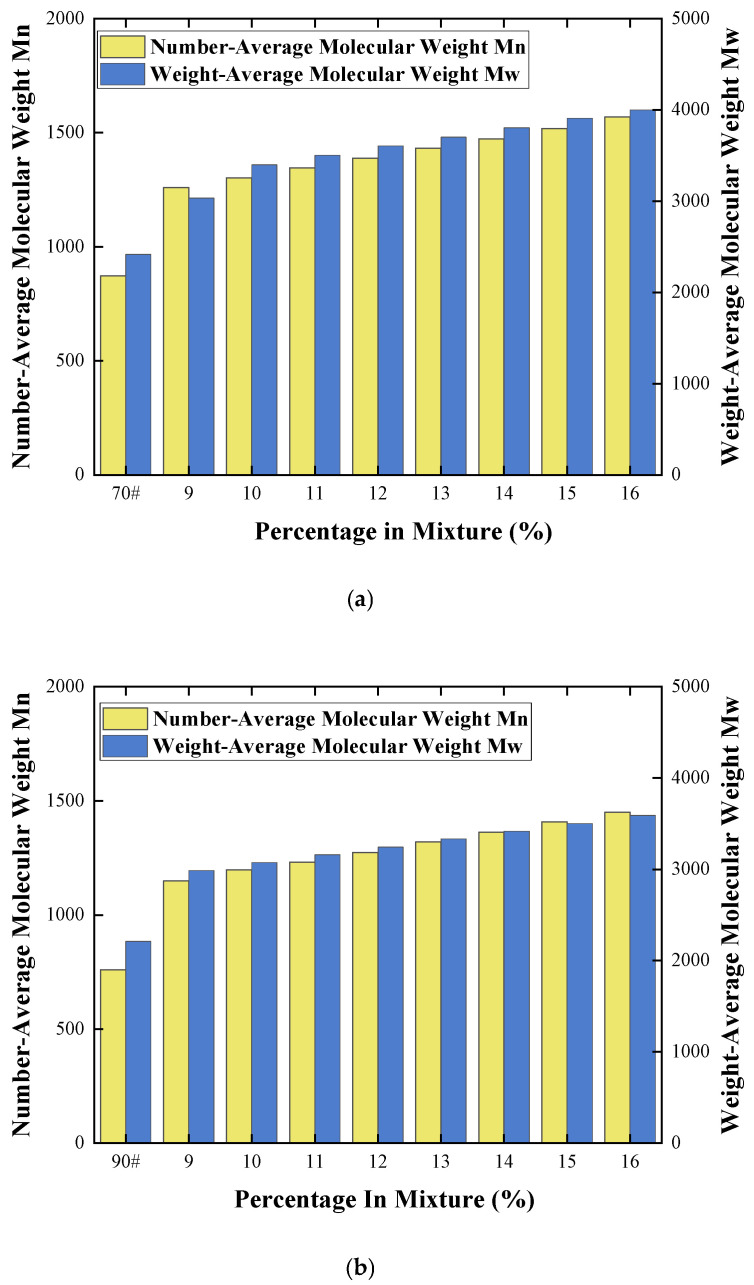
(**a**) 70# Asphalt Results from the GPC Experiment and (**b**) 90# Asphalt Results from the GPC Experiment.

The molecular weight and its distribution are intrinsic factors governing the performance of asphalt, significantly influencing its viscosity, softening point, penetration, and temperature susceptibility. To reveal the modification mechanism of COCR-modified asphalt at the molecular structural level, this study conducted GPC tests on the 90# base asphalt, 70# base asphalt, and their modifications at different COCR contents. The aim was to analyze the influences of varying COCR dosage on the molecular weight and distribution for the two selected base asphalts. The experimental results are shown in the Figure 12 below.

The molecular weight of COCR modified 70# and 90# asphalt was quantitatively determined using GPC. The variation patterns of the number-average molecular weight (Mn) and weight-average molecular weight (Mw) under different COCR dosages (9–16%) were analyzed, and the modification effects on the two base asphalts were compared.

As the COCR dosage increased from 0% to 16%, the Mn of the 70# asphalt showed a continuous upward trend. The Mn of the base asphalt was approximately 870 g/mol, rising to about 1570 g/mol at a 16% dosage. Similarly, the Mn of the 90# base asphalt was approximately 760 g/mol, increasing to about 1450 g/mol at a 16% dosage. Evidently, the addition of COCR significantly enhanced the Mn, indicating that COCR effectively increased the average chain length of the molecular chains in the base asphalt.

The Mw also showed a notable increase with the rise in COCR dosage. Taking the 70# asphalt as an example, the Mw increased from approximately 3032 g/mol at a 9% dosage to about 4000 g/mol at a 16% dosage. The growth rate of Mw was greater than that of Mn, suggesting that the addition of COCR not only increased the average molecular chain length but also promoted the formation of larger molecular components, resulting in a broadening of the molecular weight distribution.

The Mw/Mn ratio slightly increased with the rise in content, indicating that the introduction of COCR gradually broadened the molecular weight distributions of both asphalts. This phenomenon was attributed to physical adsorption and cross-linking reactions between COCR and the asphalt matrix [38].

#### 3.3.3. Four-Fraction Composition (SARA) Analysis

**Figure 13:**
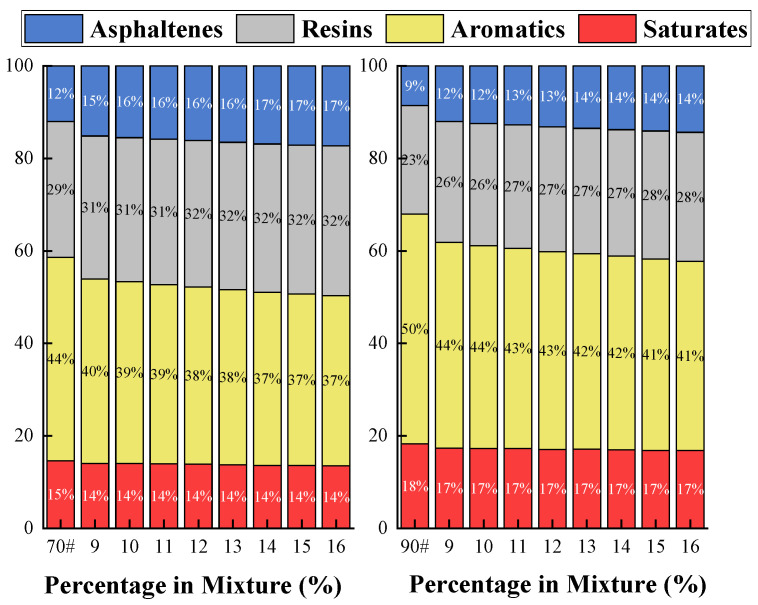
SARA Analysis Results.

The colloidal stability and macroscopic properties of asphalt are fundamentally determined by its four-fraction composition: saturates, aromatics, resins, and asphaltenes [39]. To elucidate how COCR modification alters this fundamental structure, SARA analysis was conducted on both base and modified binders. These changes in the four fractions provide direct chemical evidence for the reinforcement mechanism, thereby accounting for the observed variations in rheological behavior and engineering performance. The experimental results are shown in the Figure 13 below.

As the COCR dosage increases, the asphaltene content of both asphalt types shows a significant increase. For the 70# asphalt, the asphaltene content increases from 12% in the base asphalt to 18% at a 16% COCR dosage. The 90# asphalt exhibits a more pronounced increase, rising from 8% to 15%. This observation is attributable to the substantial proportion of heavy fractions present in COCR. Similarly, the overall increase in resin content is primarily due to resin-rich components from COCR. This enhancement in resin content is beneficial for stabilizing the colloidal structure of the asphalt, potentially improving its durability and stress-dispersing capabilities [40].

The reason for this phenomenon is that COCR is rich in heavy components found in asphalt, such as asphaltenes and resins. By introducing these components into the base asphalt, the colloidal stability of the system is enhanced. This improvement in colloidal structure contributes significantly to the asphalt’s high-temperature performance and aging resistance, as the reinforced framework better resists deformation and oxidative degradation. Furthermore, consistent with the GPC results that show a broadening of the molecular weight distribution, the components in COCR may promote the aggregation of the native resin fraction (one of the four SARA components) in the asphalt through strong physical interactions. These associated structures exhibit solubility parameters and molecular weights comparable to those of asphaltenes, leading to their reclassification as part of the asphaltene fraction in the SARA analysis, thereby increasing the measured asphaltene content [41,42].

### 3.4. Performance of COCR-Modified Asphalt Mixtures

#### 3.4.1. Mix Design for COCR-Modified Asphalt Mixtures

**Figure 14:**
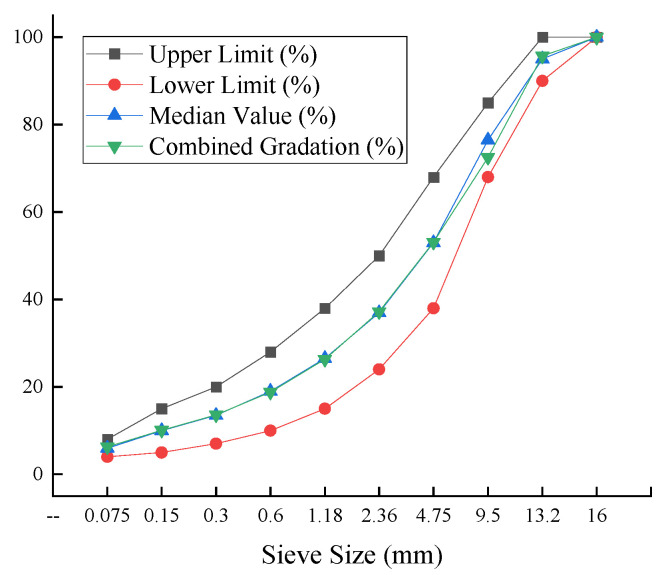
Gradation of AC-13 Asphalt Mixture.

Frictional resistance and interlocking force between mineral aggregates are key factors determining the mechanical properties and stabilities of asphalt mixtures. To systematically evaluate their influence, this study selects the continuously graded AC-13 asphalt mixture, commonly used in the upper and intermediate layers, for investigation. The gradation schemes are designed as presented in Table 7, and the corresponding gradation curves are shown in Figure 14.

#### 3.4.2. Marshall Stability

**Figure 15:**
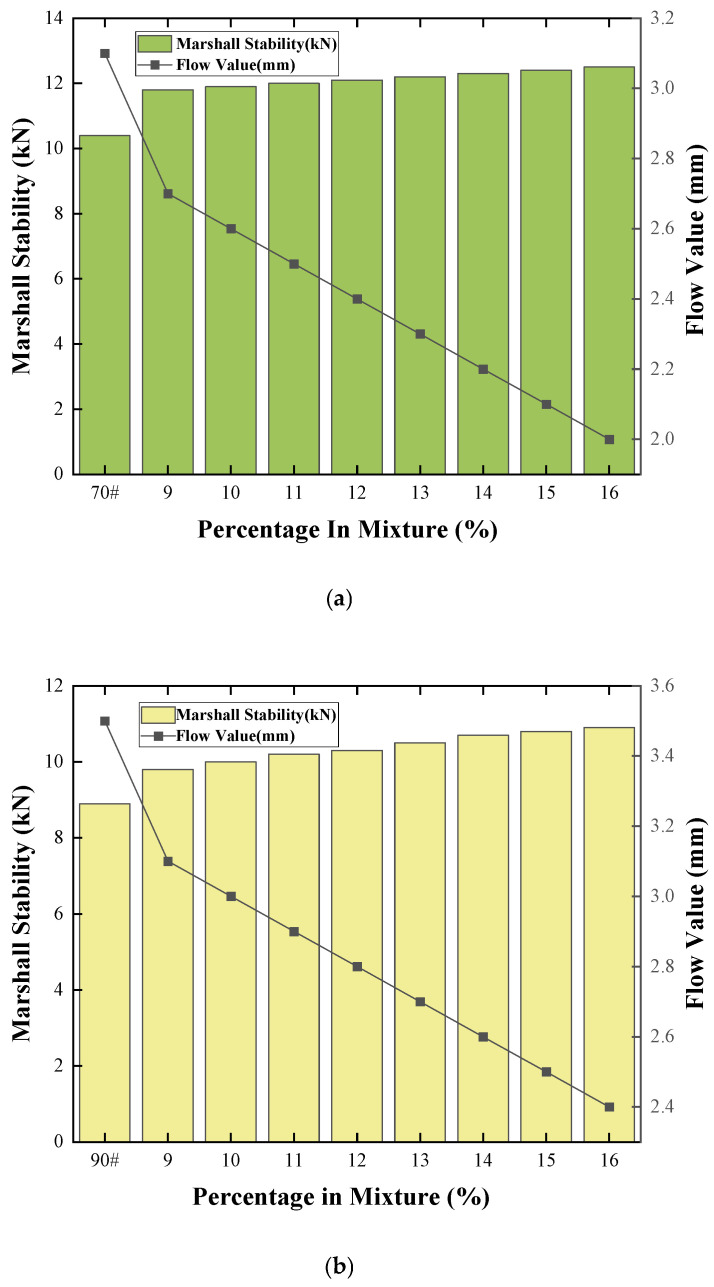
(**a**) 70# Asphalt Mixture Marshall Stability Test Results and (**b**) 90# Asphalt Mixture Marshall Stability Test Results.

The Marshall stability of an asphalt mixture is fundamental to its ability to withstand traffic loads. As a direct measure of its shear resistance and load-bearing capacity, the Marshall stability value is a key parameter in mix design and performance evaluation. The Marshall stability test results for COCR-modified asphalt mixtures are presented in Figure 15 below.

As shown in the figure above, for both base asphalts, the stability increases significantly and continuously with increasing COCR content while the flow value decreases markedly. Taking the 20% content as an example, the stability increases by 1.5 kN and 2.5 kN for the 70# and 90# mixtures, respectively, and the flow value decreases correspondingly. An increase in stability signifies enhanced high-temperature rutting resistance. This indicates that the incorporation of COCR effectively improves the high-temperature stability of the mixture. The decrease in flow value suggests an increase in mixture stiffness and a reduction in plasticity, which corroborates the conclusion drawn from the stability increase. Together, they indicate that the mixture is becoming stiffer and possesses improved high-temperature performance.

This phenomenon is attributed to the high content of asphaltenes and other heavy components in COCR, as confirmed by the SARA analysis presented in Section 3.3.3. Its addition increases the binder viscosity, hardening the overall asphalt cement. Simultaneously, the stiffer asphalt provides stronger cohesion, thereby enhancing the interlocking force and structural strength between aggregates.

Although the addition of COCR leads to identical trends in performance change (increased stability and decreased flow value) for both base asphalts, a key difference exists in the magnitude of these changes. This reveals the differential modification effect of COCR on different base asphalts. The modification effect is more pronounced for the 90# asphalt. The data trend suggests that at the same dosage, the percentage increase in stability and decrease in flow value are generally greater for the 90# asphalt compared to the 70# asphalt [43].

#### 3.4.3. Dynamic Stability

**Figure 16:**
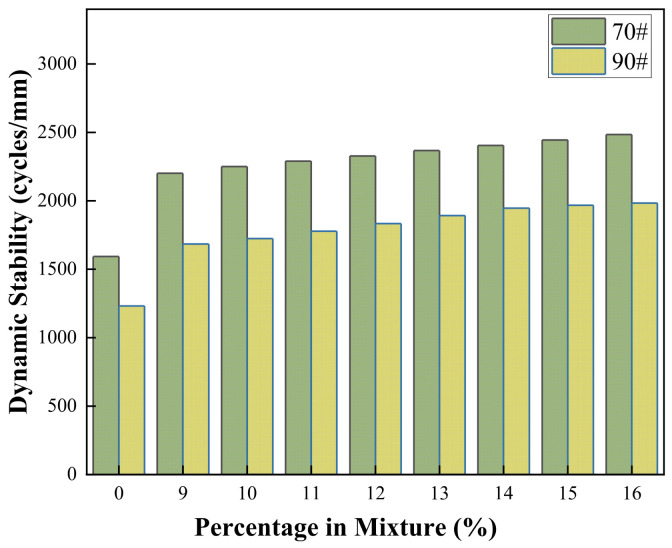
Dynamic Stability Test Results for COCR-Modified Asphalt Mixtures.

The high-temperature stability of asphalt mixture is crucial for ensuring long-term pavement performance. As a core indicator for evaluating this performance, the change in dynamic stability directly reflects the effectiveness of the modifier. The dynamic stability test results for COCR- modified asphalt mixtures are shown in the Figure 16 below.

As illustrated, the addition of COCR produces a consistent reinforcing effect on both base asphalts. For both the 70# and 90# asphalts, the dynamic stability of the mixtures exhibits a strictly monotonic increasing trend with higher COCR content. This pattern confirms that COCR effectively enhances the high-temperature rutting resistance of asphalt mixtures. The underlying reason is the hardening effect imparted by the heavy components in the residue on the asphalt binder, which increases the overall mixture stiffness and its resistance to shear deformation.

At a 10% COCR dosage, the dynamic stabilities of 70# and 90# asphalt increased by approximately 41% and 40%. For the 70# asphalt mixture, the dynamic stability increased from 1593 cycles/mm for the base material to 2250 cycles/mm with modification, representing an absolute increase of 657 cycles/mm. For the 90# asphalt mixture, the dynamic stability increased from 1232 cycles/mm to 1723 cycles/mm, with an absolute increase of 491 cycles/mm.

The reason for this phenomenon is that the 70# asphalt, due to its lower penetration grade, has a harder base binder, which gives its mixture a higher initial dynamic stability. When COCR is incorporated into the inherently stiffer 70# asphalt system, it creates a more pronounced synergistic rigidifying effect. This leads to a greater absolute performance gain and consistently superior dynamic stability levels compared to the modified 90# mixture [44].

#### 3.4.4. Residual Stability

**Figure 17:**
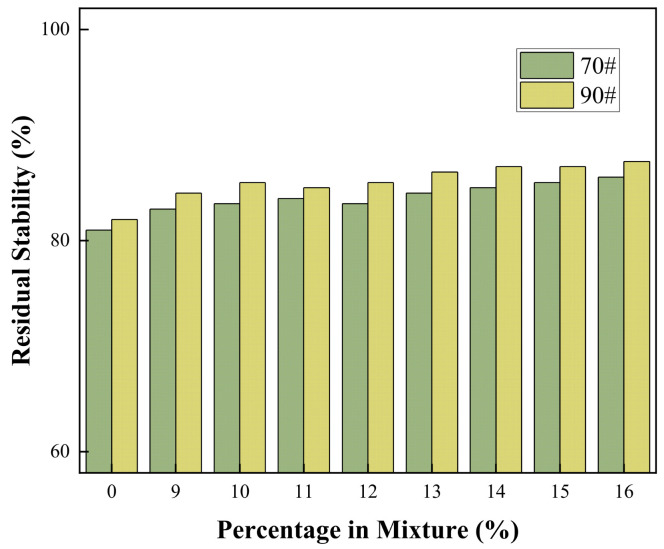
Residual Stability Test Results.

The moisture susceptibility of an asphalt mixture is a critical factor affecting pavement durability. The retained Marshall stability, serving as a key indicator to evaluate moisture sensitivity, directly quantifies the mixture’s resistance to water damage. The moisture stability test results for COCR-modified asphalt mixtures are shown in the Figure 17 below.

As shown in the figure, for both grades of base asphalt, the residual stability of their mixtures does not exhibit significant fluctuations with changes in the COCR content. Throughout the entire dosage range (9% to 16%), the measured residual stability values for both the 70# and 90# asphalt mixtures are higher than the minimum specification requirement of 85%, demonstrating excellent moisture susceptibility resistance.

This result indicates that COCR, as a modifier, exhibits good compatibility with both grades of base asphalt. Its incorporation does not disrupt the stability of the asphalt colloidal structure, nor does it produce a significant negative impact on the adhesion between asphalt and aggregates. Consequently, the moisture damage resistance of the mixtures remains stable [45].

#### 3.4.5. Low-Temperature Bending Test

**Figure 18:**
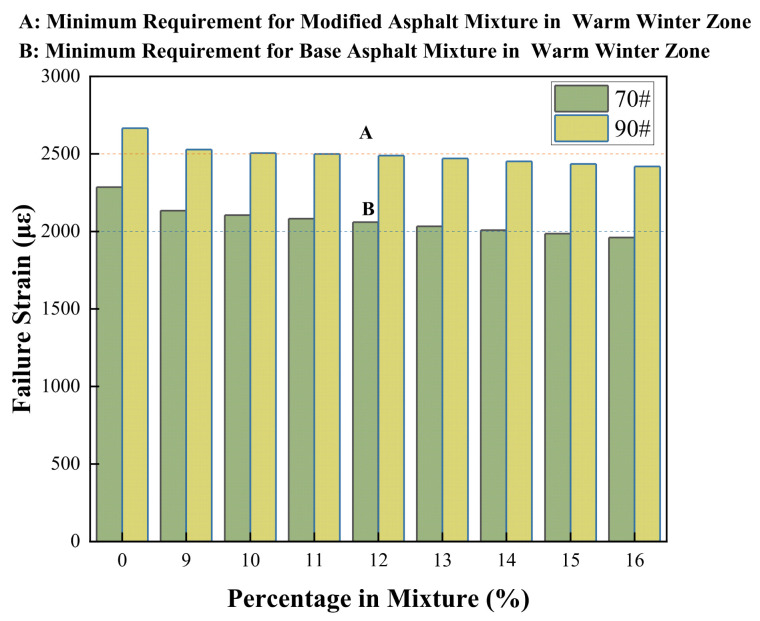
Low-Temperature Bending Test Results.

The low-temperature bending test in this study was conducted at −10 °C. The test results directly reflect the deformation capacity and brittle fracture characteristics of the mixture under low-temperature conditions, providing important guidance for evaluating the pavement’s resistance to thermal shrinkage cracks and reflective cracks during cold seasons. By preparing beam specimens with dimensions of 250 mm (length) × 30 mm (width) × 35 mm (height), the load and mid-span deflection data were collected in real time during the testing process, ultimately calculating and analyzing the flexural stiffness modulus as a core evaluation index. The results of the low-temperature bending test are presented in the Figure 18 below.

The reduction in failure strain is more pronounced for the 70# asphalt mixture than for the 90# mixture, indicating that the low-temperature performance of the 70# mixture is more sensitive to the COCR content. At a 16% dosage, the failure strain of the 70# mixture is approximately 86% of its baseline value, whereas that of the 90# mixture is about 90% of its baseline value. From the perspective of performance retention rate, the 90# asphalt mixture demonstrates slightly better stability.

The decrease in failure strain directly indicates a reduction in the low-temperature deformation capacity and crack resistance of the asphalt mixture. The addition of COCR likely disrupts the original colloidal structure stability of the base asphalt, leading to decreased low-temperature plasticity and increased brittleness. This is because the 70# asphalt initially presents a stiffer, more structured colloidal system. The introduction of the residue, acting as a perturbing factor, likely has a negative impact on asphalt structures. In contrast, the 90# asphalt, with its initially softer and more elastic colloidal system, exhibits slightly better tolerance to the incorporation of the residue, leading to a relatively smaller absolute magnitude and rate of performance degradation [46].

Within the dosage range of this study (9% to 16%), COCR is a detrimental factor for the low-temperature crack resistance of asphalt mixtures. Its incorporation leads to a decrease in the mixture’s failure strain, increasing the risk of low-temperature cracking in pavements. However, according to specification requirements, mixtures with 10% COCR in 90# asphalt still meet the requirements for regions with hot summers and warm winters.

## 4. Conclusions

This study systematically evaluated the feasibility of utilizing COCR as a modifier for asphalt binders. A comprehensive experimental program was conducted, including conventional performance testing, rheological characterization, and microstructural analysis, to investigate the effects of COCR on the properties of 70# and 90# base asphalts. The main findings are summarized as follows:

(1) High-Temperature Performance Enhancement: The incorporation of COCR significantly improves the high-temperature performance of both 70# and 90# base asphalts. This is evidenced by the increased softening point, enhanced rutting factor (G*/sinδ) from temperature sweep tests, improved dynamic stability of mixtures, and greater elastic recovery in MSCR tests. The improvement exhibits a positive correlation with a COCR content of up to 16%.
(2) Low-Temperature Performance Trade-off: The enhancement in high-temperature performance comes at the expense of reduced low-temperature crack resistance. Both base asphalts show decreased ductility and failure strain in bending tests with increasing COCR content. The 70# asphalt mixture demonstrates greater sensitivity to COCR modification in terms of low-temperature performance deterioration compared to the 90# asphalt.
(3) Microstructural Modification Mechanism: FTIR, GPC, and SARA analyses collectively reveal that COCR modification primarily alters the asphalt’s colloidal structure. The increase in asphaltene content, broadening of molecular weight distribution, and shift from a sol–gel structure to a gel-type structure contribute to the performance changes. These modifications enhance high-temperature stability but reduce low-temperature flexibility.
(4) Optimal Dosage Recommendation: Considering the balance between high-temperature improvement and low-temperature performance reduction, the recommended COCR content is 10% for 90# base asphalt for application in regions with hot summers and warm winters. These dosages provide significant high-temperature enhancement while maintaining adequate low-temperature performance that meets specification requirements.

In summary, COCR is an effective modifier for producing asphalt binders with superior high-temperature performance, offering a promising approach for the resourceful utilization of this industrial residue. The addition of COCR increases the complex shear modulus, rutting factor, and elastic recovery while reducing non-recoverable creep compliance, as confirmed by MSCR tests. Storage stability remains acceptable for all modified binders. FTIR and SARA analyses reveal that the reinforcement mechanism is primarily attributed to the introduction of asphaltenes and resins from COCR, which strengthen the colloidal structure of the asphalt. Although low-temperature performance is compromised, the overall balance of properties suggests that 10% COCR in 90# base asphalt is suitable for hot-summer and warm-winter regions.

Future research should focus on optimizing the modification process, exploring methods to mitigate the negative impact on low-temperature performance, systematically evaluating the influences of different aggregate types (including variations in mineralogy, shape, and surface properties) on the performance levels of COCR modified asphalt mixtures, and conducting long-term field performance evaluations to facilitate the practical application of COCR-modified asphalt in pavement engineering projects.

## Figures and Tables

**Table 1:** Basic Properties of the Coal–Oil Co-Processing Residue.

Technical Indicator		Test Result	GB 4284-2018 [27]
Color		Black	-
Water Content (%)		0.4	-
Solubility in Trichloroethylene (%)		89.1	-
Heavy Metal Content	Pb (mg/kg)	21.2	300
	Cd (mg/kg)	0.4	3
	Cr (mg/kg)	36.8	500
	Hg (mg/kg)	0.01	3
	As (mg/kg)	6.5	30

**Table 2:** Elemental Composition of the Coal–Oil Co-Processing Residue.

Elemental Content (ω), %	Data
C	59.6
H	4.3
S	6.1
N	0.5
O	18.1

**Table 3:** Basic Properties of 70# Base Asphalt and 90# Base Asphalt.

Technical Indicator		70# Test Result	Technical Requirement	90# Test Result	Technical Requirement
Penetration (25 °C, 0.1 mm)		77	60–80	96	80–100
Ductility (15 °C, cm)		>100	≥100	>100	≥100
Softening Point (°C)		48	≥45	45	≥44
Wax Content (%)		1.5	<2.2	1.7	<2.2
Solubility (%)		99.9	≥99.5	99.9	≥99.5
Dynamic Viscosity (60 °C, Pa·s)		217	≥160	182	≥160
Flash Point (°C)		265	≥260	258	≥260
Rolling Thin Film Oven Test (RTFOT)	Mass Change (%)	−0.2	±0.8	−0.2	±0.8
	Residual Ductility (10 °C, cm)	9	≥6	12	≥6
	Penetration Ratio (%)	67	≥61%	63	≥61%

**Table 4:** Technical Properties of Coarse Aggregate.

Test Item		Test Result			Specification Requirement	Test Method
		10–15 mm	5–10 mm	3–5 mm		
Crushed Value/%		17.8	17.8	17.8	≤26	T 0316-2005
Los Angeles Abrasion Loss/%		20.2	20.2	20.2	≤28	T 0317-2005
Apparent Relative Density		2.831	2.812	2.810	≥2.6	T 0330-2005
Bulk Relative Density		2.772	2.765	2.763	Measured	-
Water Absorption/%		0.66	0.51	0.54	≤2.0	-
Adhesiveness to Asphalt/Grade		4	4	4	≥4	T 0616-1993
Soundness/%		7.3	7.3	7.3	≤12	T 0314-2000
Flakiness & Elongation Particles Content/%	Particle > 9.5 mm	-	7.9	-	≤12	T 0312-2005
	Particle < 9.5 mm	-	8.6	-	≤18	
Soft Rock Content/%		0.2	0.2	0.2	≤3	T 0320-2000

**Table 5:** Technical Properties of Fine Aggregate.

Test Item	Test Result	Specification Requirement	Test Method
Apparent Relative Density	2.849	≥2.50	T 0330-2005
Bulk Relative Density	2.811	Measured Value	T 0330-2005
Water Absorption/%	0.77	Measured Value	T 0330-2005
Sand Equivalent/%	63	≥60	T 0334-2005
Clay Content (<0.075 mm)/%	0.87	≤3	T 0333-2000
Methylene Blue Value/(g/kg)	1.43	≤25	T 0349-2005

**Table 6:** Technical Specifications of Mineral Filler.

Test Item		Test Result	Specification Requirement	Test Method
Apparent Density (g/cm^3^)		2.831	≥2.500	T 0352-2000
Apparent Relative Density		2.842	Measured Value	T 0352-2000
Particle Size Distribution	<0.6 mm	100	100	T 0352-2000
	<0.15 mm	99.2	90~100	T 0351-2000
	<0.075 mm	95.1	75~100	T 0351-2000
Hydrophilic Coefficient		0.7	<1.0	T 0353-2000

**Table 7:** Gradation Design of AC-13 Mixture.

Sieve Size (mm)	16	13.2	9.5	4.75	2.36	1.18	0.6	0.3	0.15	0.075
Upper Limit (%)	100	100	85	68	50	38	28	20	15	8
Lower Limit (%)	100	90	68	38	24	15	10	7	5	4
Median Value (%)	100	95	76.5	53	37	26.5	19	13.5	10	6
Combined Gradation (%)	100	95.7	72.5	53.1	37.2	26.3	18.8	13.6	10.1	6.3

## Data Availability

The original contributions presented in this study are included in the article. Further inquiries can be directed to the corresponding authors.
